# Acute post-disaster medical needs of patients with diabetes: emergency department use in New York City by diabetic adults after Hurricane Sandy

**DOI:** 10.1136/bmjdrc-2016-000248

**Published:** 2016-07-26

**Authors:** David C Lee, Vibha K Gupta, Brendan G Carr, Sidrah Malik, Brandy Ferguson, Stephen P Wall, Silas W Smith, Lewis R Goldfrank

**Affiliations:** 1 Ronald O. Perelman Department of Emergency Medicine, New York University School of Medicine, New York, New York, USA; 2 Department of Population Health, New York University School of Medicine, New York, New York, USA; 3 Department of Emergency Medicine, Sidney Kimmel Medical College, Thomas Jefferson University, Philadelphia, Pennsylvania, USA; 4 Department of Health & Human Services, Emergency Care Coordination Center, Office of the Assistant Secretary for Preparedness & Response, Washington, DC, USA

**Keywords:** Emergency Medicine, Population Health, Public Health/Surveillance

## Abstract

**Objective:**

To evaluate the acute impact of disasters on diabetic patients, we performed a geospatial analysis of emergency department (ED) use by New York City diabetic adults in the week after Hurricane Sandy.

**Research design and methods:**

Using an all-payer claims database, we retrospectively analyzed the demographics, insurance status, and medical comorbidities of post-disaster ED patients with diabetes who lived in the most geographically vulnerable areas. We compared the patterns of ED use among diabetic adults in the first week after Hurricane Sandy's landfall to utilization before the disaster in 2012.

**Results:**

In the highest level evacuation zone in New York City, postdisaster increases in ED visits for a primary or secondary diagnosis of diabetes were attributable to a significantly higher proportion of Medicare patients. Emergency visits for a primary diagnosis of diabetes had an increased frequency of certain comorbidities, including hypertension, recent procedure, and chronic skin ulcers. Patients with a history of diabetes visited EDs in increased numbers after Hurricane Sandy for a primary diagnosis of myocardial infarction, prescription refills, drug dependence, dialysis, among other conditions.

**Conclusions:**

We found that diabetic adults aged 65 years and older are especially at risk for requiring postdisaster emergency care compared to other vulnerable populations. Our findings also suggest that there is a need to support diabetic adults particularly in the week after a disaster by ensuring access to medications, aftercare for patients who had a recent procedure, and optimize their cardiovascular health to reduce the risk of heart attacks.

**Key messages**

- Elderly diabetic adults are at high risk for requiring postdisaster emergency care.
- There is need to support diabetic patients in the first week after a disaster.
- Specific medical issues such as myocardial infarction increase among diabetic adults in the days after a disaster.

## Introduction

On 29 October 2012, Hurricane Sandy made landfall on the East Coast of the USA, causing severe storm surges and immense destruction.^1^ In New York City alone, 305 000 homes were destroyed, and the city sustained 19 billion dollars in damage including to critical health facilities and infrastructure.^2^ There were 159 deaths; of which, 72 were directly attributed to the storm and 87 were indirectly due to the storm's impact, ie, extended power outages which led to hypothermia, falls in the dark by the elderly, or carbon monoxide poisoning from improperly used generators and cooking devices.^3^

Although the acute impact on mortality and cost of destruction was well estimated for Hurricane Sandy, less is known about the acute impact this disaster had on patients with chronic diseases such as diabetes.^3^ ^4^ Patients with diabetes are a particularly vulnerable population during a disaster. Disrupted access to care, inability to monitor glucose, poor nutrition, limited physical activity, damaged or lost medications, inability to safely store insulin due to power loss, and lack of prescription refills all place diabetic adults at increased risk for developing acute postdisaster medical needs, and potential morbidity or mortality in the days following a disaster.^5^ ^6^

There is a paucity of information on the acute impact of disasters on diabetic patients, specifically following Hurricane Sandy. Longer term studies in the literature from other disasters have demonstrated poor glycemic control as measured by HbA1c months later and poorer quality of life both thought to be attributable to the increased physical and emotional stress experienced by diabetic patients.^7^ ^8^ However, in our recent study, screening for conditions that demonstrated increased postdisaster use of emergency care, we found that there was also a statistically significant increase in the number of New York City emergency department (ED) patients presenting with a primary or secondary diagnosis of diabetes even in the first week after Hurricane Sandy.^9^ These findings require additional investigation as to which characteristics make certain diabetic patients at higher risk for needing acute medical care after a disaster.

The goal of this study was to identify salient patient characteristics that increase the likelihood that a diabetic patient will develop acute medical needs after a disaster such as Hurricane Sandy. We analyzed data on patients over the age of 18 years focusing on those who lived in the highest risk evacuation zone in New York City. We investigated patient demographics, insurance status, and medical comorbidities that were associated with increased ED use among these diabetic adults. This study provides insight into how to support diabetic populations in future disasters and to develop a framework for targeted interventions and deployment of disaster response resources.

## Research design and methods

### Study design

Using an all-payer claims database of ED visits in New York City for 2012, we compared the demographic characteristics, insurance status, geographic distribution, and health conditions of ED patients with a primary versus secondary diagnosis of diabetes before and after landfall of Hurricane Sandy. To evaluate post-disaster ED utilization by diabetic adults that occurred in geographically vulnerable regions, we compared ED use for the first week after the disaster to baseline ED use in 2012 prior to 29 October the day on which Hurricane Sandy struck the East Coast of the USA.

### Data source

The New York State Department of Health collects claims data from hospitals on ED visits and inpatient hospitalizations. The Statewide Planning and Research Cooperative System (SPARCS) is the most comprehensive resource for ED utilization in New York State.^10^ It includes privately insured, Medicare, Medicaid, and uninsured patients. In addition, to patient demographic data and insurance status, SPARCS includes diagnosis codes and patient addresses that can be used to identify health conditions and the exact location of a patient's residence.

### Study population

Our study included all adult patients aged 18 years and older who visited a New York City ED in 2012, had a home address located in New York City, and had either a primary or secondary diagnosis of diabetes. We excluded patients from correctional facilities and nursing homes, and patients who visited an ED associated with a specialty hospital (ie, surgical subspecialty, oncological, or Veterans Administration facilities). These exclusions were performed so that we studied non-institutionalized New York City adults with diabetes who visited a 911-receiving ED based at a general acute care hospital in New York City.

### ICD-9 diagnosis codes

To identify ED patients with a primary or secondary diagnosis of diabetes, we scanned International Classification of Diseases, 9th Edition (ICD-9) codes for a prefix of 250 (ie, 250.00–250.93), which includes diabetes diagnoses with and without complications. In a study of patients at the University of Pittsburgh Medical Center, the presence of a diabetes diagnosis code in ED records was 95% sensitive and 99% specific for identifying diabetic adults among ED patients.^11^ In fact, ED data had higher accuracy than outpatient records (79% sensitive, 98% specific), inpatient records (84% sensitive, 97% specific), and identification methods based on scanning pharmacy records for diabetes medications and laboratory tests for elevated HgbA1c or blood glucose concentrations.^11^

In addition, to identify health conditions associated with ED visits for patients with a primary or secondary diagnosis of diabetes, we also evaluated the frequency of other ICD-9 codes based on the first three-digit and/or letter prefix. For patients who had an ED visit with a primary diagnosis of diabetes, we evaluated the other secondary diagnosis codes to evaluate other conditions and comorbidities that may have contributed to increases in ED use after the storm. For patients who had a secondary diagnosis of diabetes, we evaluated the primary diagnosis code associated with the ED visit to evaluate the presenting cause of ED visits among patients who have a history of diabetes, but came to the ED for other conditions.

### Evacuation zones

In our prior study of ED utilization patterns, we had found that adults with a history of diabetes presented in significantly increased numbers in the level 1 evacuation zone. In this study, we focused on this subset of diabetic adults by studying diabetic patients located in the level 1 evacuation zone (at the highest risk).^9^ These revised evacuation zones were developed by the New York City Office of Emergency Management in response to Hurricane Sandy's impact and were generally areas of the city most impacted by the storm. To identify these patients, we geocoded the addresses of diabetic adults in New York City and analyzed the sample of patients whose address was located in the level 1 evacuation zone based on a publicly available geographic shapefile called ‘Atomic Polygons’ (Release 15B, April 2015), which is available from the New York City Department of City Planning.^12^

### Statistical analyses

We first evaluated the geographic distribution of ED patients from any part of New York City who presented with a primary or secondary diagnosis of diabetes by comparing the number of ED visits for the week after the storm compared to weekly baseline ED use in 2012 prior to landfall of Hurricane Sandy.^13^ We analyzed this geographic distribution by postal ZIP code to identify areas of significant changes in ED use. We calculated a Z-score for the number of ED visits for the week after the storm's landfall using an average and SD from the pre-disaster weeks in 2012. Then, we identified areas with significant changes in ED use by mapping ZIP codes with a Z-score of 1.645, 1.96, and 2.545, which we noted as a 90%, 95%, and 99% confidence of an increase or decrease in postdisaster ED use.

We then examined the demographic characteristics and insurance status of ED patients with a primary or secondary diagnosis of diabetes focusing on patients living in the level 1 evacuation zone. To compare these characteristics for the week after the storm's landfall to baseline utilization patterns, we analyzed the average weekly proportion of elderly (aged 65 years and older), female, non-Hispanic black, and Hispanic patients, in addition to the proportion of privately insured, Medicare, Medicaid, and uninsured patients in 2012 prior to Hurricane Sandy's landfall. For these weeks in 2012 prior to the disaster, we computed an average and SD and then calculated Z-scores for patient characteristics the week after the storm to determine statistically significant changes using a p value of <0.05.

Finally, to analyze the other primary and secondary diagnoses, we determined the most common primary diagnoses among ED patients with a secondary diagnosis of diabetes and also the most common secondary diagnoses among ED patients with a primary diagnosis of diabetes. This analysis also focused on patients from the level 1 evacuation zone. We compared the top 10 of these primary and secondary diagnoses before and after the disaster, and we also evaluated which of these diagnoses had the highest absolute increases in the number of ED patients in the week following Hurricane Sandy's landfall compared to the pre-disaster weekly average. To be considered, the increase in ED visits for a given primary or secondary diagnosis had to be statistically significant, meaning that the Z-score for the number of ED visits for the week after storm's landfall had to be above 2.81, correlating to a p value of at least 0.005 based on a Bonferroni adjustment for comparisons among the top 10 diagnoses.

Statistical analyses were performed using Stata V.12.1 (StataCorp: College Station, Texas, USA, 2011). Geographic analysis was performed using ArcGIS Desktop 10.2 (ESRI: Redlands, California, USA, 2013).

## Results

### Study population

In evacuation zone level 1 in New York City, there were an average of 30.9 ED visits with a primary diagnosis of diabetes and 258.8 ED visits with a secondary diagnosis of diabetes during the weeks in 2012 before Hurricane Sandy's landfall (table 1). For the week after the landfall, there were statistically significant increases with 60 ED visits for a primary diagnosis of diabetes and 420 ED visits for a secondary diagnosis of diabetes in the level 1 evacuation zone (p<0.01).

**Table 1:** Characteristics of ED users with diabetes in New York City evacuation zone level 1 before and 1 week after Hurricane Sandy's landfall in 2012

Patient characteristics	Before Hurricane Sandy landfall		After Hurricane Sandy landfall		p Value for difference
	Weekly average 2012	Proportion of patients (%)	One week after the landfall	Proportion of patients (%)	
*Primary diagnosis of diabetes*					
Total	30.9	100	60	100	<0.01*
Demographics					
Elderly (aged 65 years and older)	9.8	32	26	43	0.16
Female	12.8	41	36	60	0.05
Black	12.7	41	4	27	0.09
Hispanic	6.1	20	13	22	0.76
Insurance					
Private	3.6	12	4	7	0.42
Medicare	12.7	41	33	55	0.04*
Medicaid	10.7	35	17	28	0.48
Self-Pay	3.9	12	6	10	0.69
*Secondary diagnosis of diabetes*					
Total	258.8	100	420	100	<0.01*
Demographics					
Elderly (aged 65 years and older)	128.4	50	250	60	<0.01*
Female	143.5	55	238	57	0.69
Black	73.9	29	101	24	0.19
Hispanic	50.9	20	81	19	0.92
Insurance					
Private	32.0	12	37	9	0.11
Medicare	145.9	56	279	66	<0.01*
Medicaid	67.8	26	75	18	0.02*
Self-Pay	13.1	5	29	7	0.16

ED, emergency department.

* = p-value < 0.05

Comparing the proportion of these patients to the average weekly baseline in New York City in 2012, there was a statistically significant increase in the proportion of Medicare patients presenting to an ED with a primary diagnosis of diabetes (from 41% to 55%, p=0.04). Among ED patients with a secondary diagnosis of diabetes, the proportion of Medicare patients also increased (from 56% to 66%, p<0.01). In addition, there was an increase in the proportion of elderly patients presenting with a secondary diagnosis of diabetes (from 50% to 60%, p<0.01).

**Figure 1:**
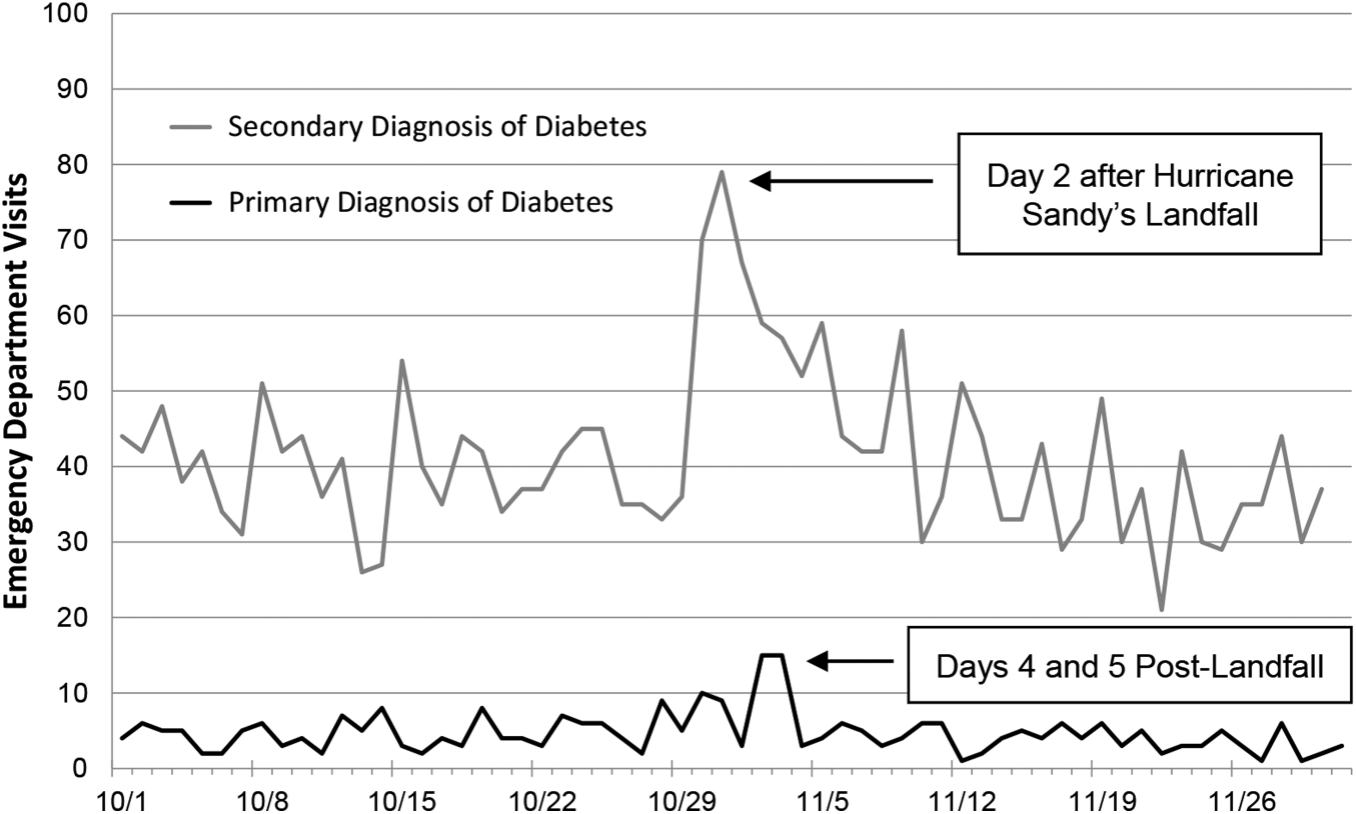
Daily changes in ED use compared to baseline utilization in October and November 2012 for patients with diabetes in New York City evacuation zone level 1. ED, emergency department. Changes in daily ED visits for a primary versus secondary diagnosis of diabetes in New York City evacuation zone level 1. The highest numbers of ED visits occur on days 4 and 5 postlandfall for a primary diagnosis of diabetes and on day 2 postlandfall for a secondary diagnosis of diabetes. ED, emergency department.

We did not find evidence of any statistically significant changes in the proportion of female, non-Hispanic black, Hispanic, privately insured, or uninsured patients presenting with a primary or secondary diagnosis of diabetes. However, among ED patients with a secondary diagnosis of diabetes, there was a statistically significant decrease in the proportion of Medicaid patients (from 26% to 18%, p=0.02) (figure 1).

### Geographic distribution

**Figure 2:**
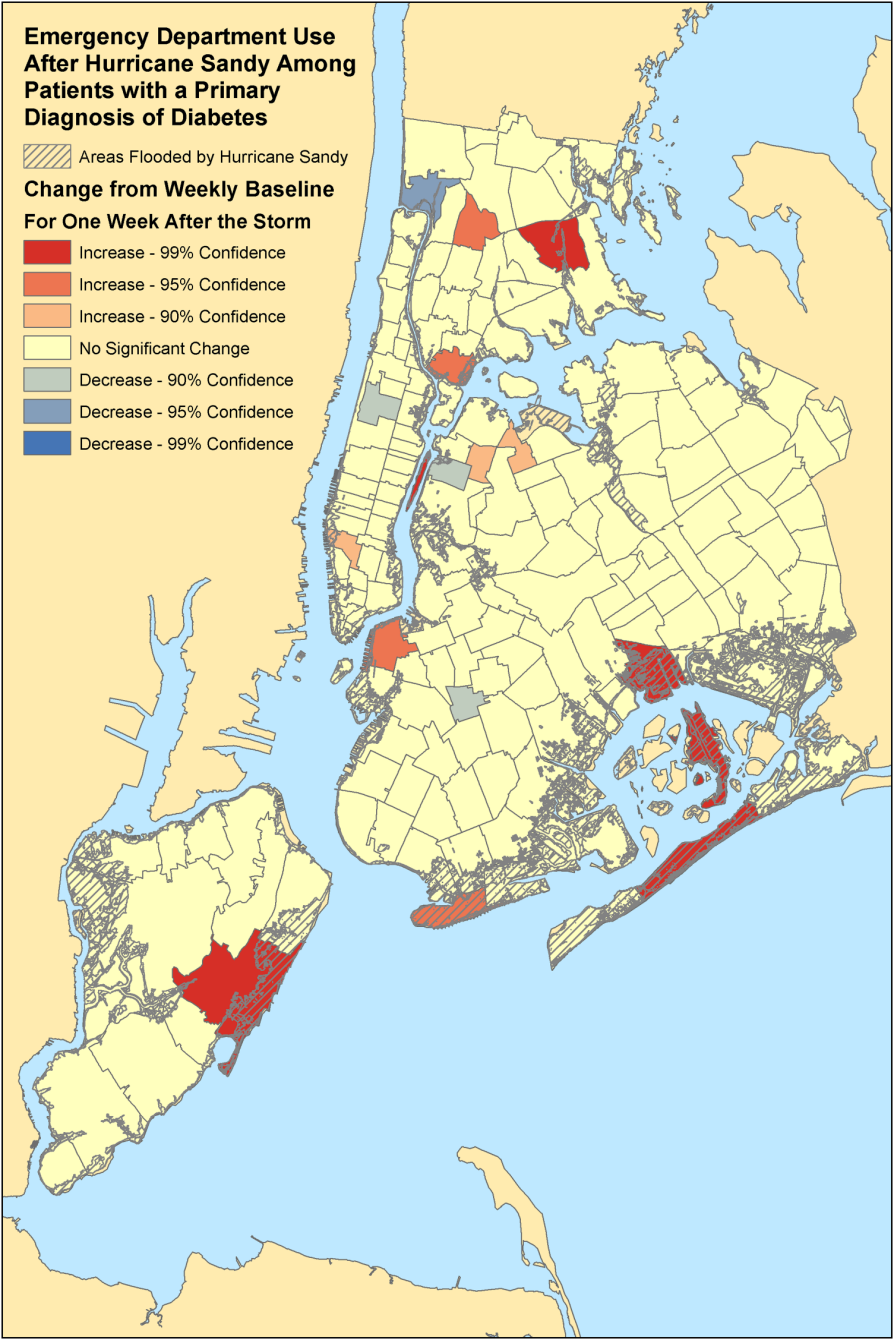
Geographic areas with increased ED visits after Hurricane Sandy by diabetic adults. Significant changes in ED visits among patients with a primary diagnosis of diabetes. Compares the week after Hurricane Sandy's landfall to baseline weekly data by New York City ZIP Codes in 2012. Flooded areas based on the FEMA Modeling Task Force Hurricane Sandy Impact Analysis. ED, emergency department.

**Figure 3:**
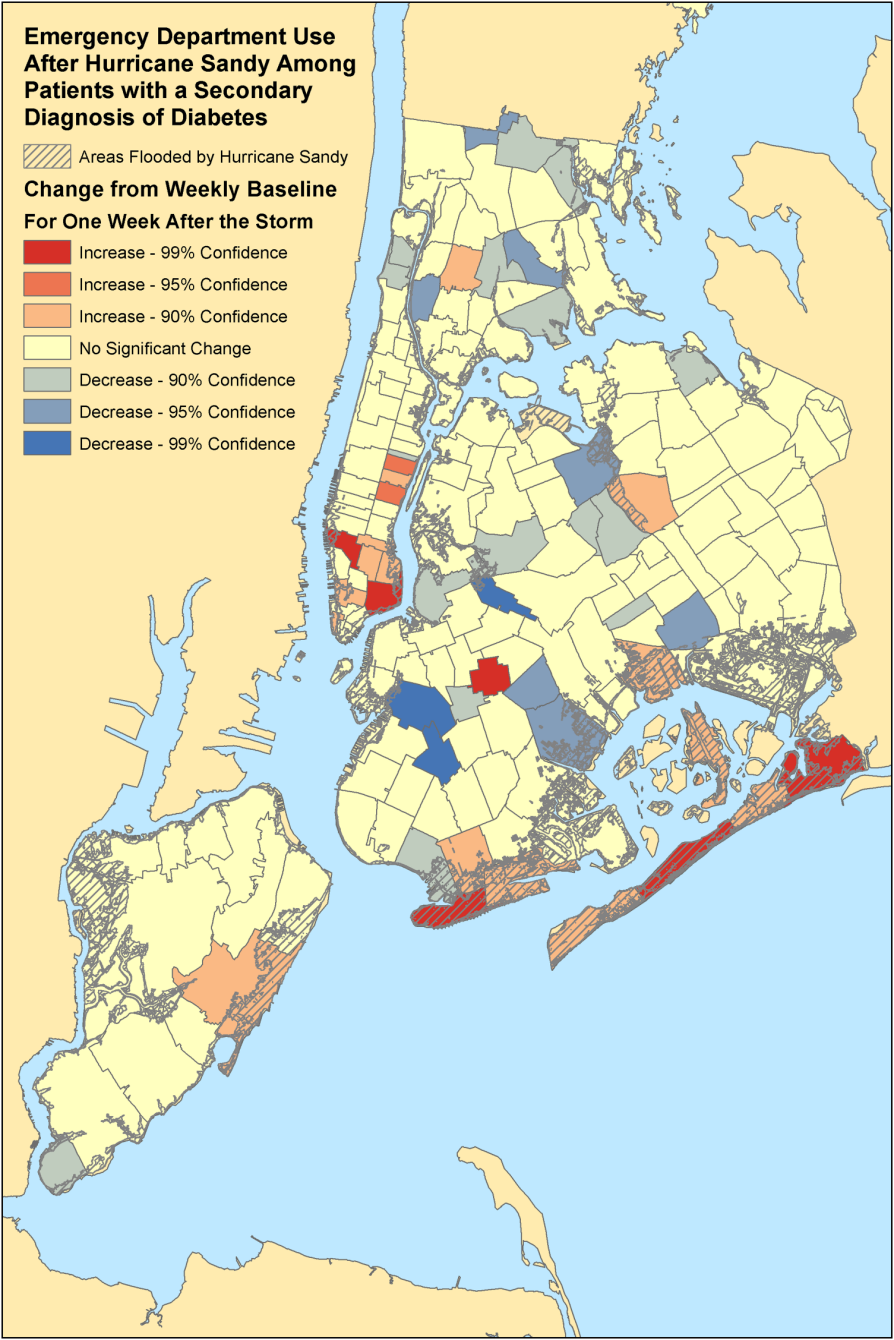
Significant changes in ED visits among patients with a secondary diagnosis of diabetes. ED, emergency department.

In our evaluation of ED patients from all regions of New York City, we identified ZIP codes with a statistically significant change in the number of ED visits by diabetic adults comparing the week after the landfall of Hurricane Sandy to the average weekly baseline prior to disaster in 2012 (figures 2 and 3). There were significant increases in ED use for a primary diagnosis of diabetes, especially, in flood-prone areas. These regions included the Rockaways (11693, 11694), Howard Beach (11414), Coney Island (11224), eastern Staten Island (10306), and parts of Brooklyn (11201) and the Bronx (10454, 10461). For a secondary diagnosis of diabetes, there was similarly a statistically significant increase in ED use in the Rockaways (11691, 11694) and Coney Island (11224), but also some increases in flood-prone areas of Manhattan (10002, 10011).

### Primary diagnoses

Among patients in the level 1 evacuation zone, the top 10 primary diagnoses among ED patients with a secondary diagnosis of diabetes for the week after Hurricane Sandy's landfall and the pre-Hurricane Sandy average weekly baseline in 2012 are shown in table 2. Comparatively, the lists are relatively similar with over half of the diagnoses appearing before and after Hurricane Sandy's landfall. Notable exceptions included a statistically significant increase in the number of myocardial infarctions (an increase from 4 to 12 cases per week in evacuation zone level 1), hypertension (an increase from 4 to 15 cases per week), chronic bronchitis (an increase from 4 to 12 cases per week), and hypertensive kidney disease (an increase from 2 to 10 cases per week). In addition, we also found statistically significant increases in the number of ED visits with a primary diagnosis for chronic kidney disease, prescription refills, drug dependence, dialysis dependence, and electrolyte disorders (p<0.005).

**Table 2:** Most common primary diagnoses of patients with a secondary diagnosis of diabetes who presented for emergency care before and 1 week after Hurricane Sandy's landfall in 2012

Weekly baseline in 2012 before Hurricane Sandy's landfall	One week after Hurricane Sandy's landfall	Highest increases before and after
Respiratory symptoms (16)	General symptoms (25)	General symptoms (+13)
General symptoms (12)	Respiratory symptoms (19)	Hypertension (+11)
Heart failure (10)	**Hypertension** (15)	Myocardial infarction (+8)
Sepsis (7)	Heart failure (13)	Hypertensive kidney disease (+8)
**Cellulitis or abscess** (7)	**Myocardial infarction** (12)	Chronic bronchitis (+8)
**Urinary tract infection** (7)	**Chronic bronchitis** (12)	Chronic kidney disease (+7)
Asthma (7)	Asthma (12)	Prescription refills (+7)
**Abdominal/pelvic symptoms** (6)	**Hypertensive kidney disease** (10)	Drug dependence (+7)
**Cardiac dysrhythmias** (5)	Sepsis (9)	Dialysis dependence (+6)
Ischemic heart disease (5)	Ischemic heart disease (9)	Electrolyte disorder (+6)

Diagnoses in bold highlight the differences in categories before and after Hurricane Sandy's landfall.

### Secondary diagnoses

We also analyzed the top 10 secondary diagnoses among ED patients in the highest level evacuation zone with a primary diagnosis of diabetes to assess associated health conditions and comorbidities (see table 3). Comparing the week after Hurricane Sandy's landfall to the predisaster average weekly baseline for 2012, a majority of the secondary diagnoses overlap before and after the storm. Notable exceptions included a statistically significant increase in the number of ED patients presenting primarily with diabetes who also had a secondary diagnosis code for a postprocedural state (an increase from 3 to 10 cases per week in evacuation zone level 1) and a secondary diagnosis code for overweight or obesity (an increase from 2 to 7 cases per week). In addition, we also found statistically significant increases in the number of ED visits with a secondary diagnosis for cardiac dysrhythmia, chronic airway obstruction, and osteomyelitis.

**Table 3:** Most common secondary diagnoses of patients presenting for EDs for a primary diagnosis of diabetes before and 1 week after Hurricane Sandy's landfall in 2012

Weekly baseline in 2012 before Hurricane Sandy's landfall	One week after Hurricane Sandy's landfall	Highest increases before and after
Hypertension (13)	Hypertension (30)	Hypertension (+17)
Hyperlipidemia (7)	Post-procedural aftercare (17)	Post-procedural aftercare (+11)
Post-procedural aftercare (6)	Chronic skin ulcer (14)	Chronic skin ulcer (+9)
Electrolyte disorder (6)	Electrolyte disorder (13)	Electrolyte disorder (+7)
Ischemic heart disease (5)	Hyperlipidemia (11)	Post-procedural state (+7)
Chronic skin ulcer (5)	**Post-procedural state** (10)	Overweight or obese (+5)
Chronic kidney disease (4)	Ischemic heart disease (10)	Ischemic heart disease (+5)
**Hypertensive kidney disease** (4)	Drug abuse (8)	Cardiac dysrhythmia (+4)
Drug abuse (4)	Chronic kidney disease (7)	Chronic airway obstruction (+4)
**Other health hazards (7)**	**Overweight or obese (7)**	Osteomyelitis (+4)

Diagnoses in bold highlight the differences in categories before and after Hurricane Sandy's landfall.

ED, emergency department.

## Conclusions

Prior literature has documented the longer term impact of disasters on diabetic adults. Hurricane Sandy was the second most destructive hurricane in US history, only surpassed by Hurricane Katrina in 2005.^14^ After Hurricane Katrina, there was evidence of long-term effects of the disaster on diabetic patients when glycemic control was measured by HbA1c and quality of life was assessed.^7^ ^8^ ^15^ ^16^ In studies of international disasters, there has similar been evidence of disruptions in glycemic control and worse quality of life among individuals affected by events like earthquakes in Japan, Italy, and Turkey.^17–22^ The presumed mechanism of impact is thought to be mediated by the stress caused by the disaster, which has also been seen in other non-natural disasters.^23–25^ Our study expands this body of literature by specifically analyzing the acute impact of disasters on diabetic patients by evaluating ED use by diabetic adults after Hurricane Sandy.

In our geographic analysis, we found that diabetic adults in evacuation zone level 1 were more likely to have increased the need for post-disaster emergency care. We also found that elderly diabetic adults aged 65 years and older and Medicare patients are particularly at risk for requiring postdisaster emergency care compared to other vulnerable diabetic populations. Finally, through our analysis of diagnosis codes, we found that certain coexisting medical conditions and comorbidities place diabetic patients at higher risk for requiring acute medical care in the week immediately following a disaster such as Hurricane Sandy.

There are a unique constellation of factors that combine to increase vulnerability among elderly patients to the negative effects of catastrophic events.^26^ ^27^ Many elderly patients depend on complex medication regimens or medical equipment.^28^ Some are dependent on caregivers to help manage their lives, and with disaster-related disruptions, these caregivers may be unable to care for this population.^29^ Complicating these risk factors, these patients may not be adequately prepared for disasters as at least one study shows that only 31% of elderly had a disaster plan in place.^30^ Finally, the intersection between multiple chronic diseases and advanced age can push those at the margins of compensated chronic disease into decompensated states.^31^

We analyzed the coexisting medical conditions and comorbidities to identify those that were associated with increased ED use the week after Hurricane Sandy's landfall compared to the pre-disaster average weekly baseline in 2012 among diabetic patients from the highest risk evacuation zone.^9^ For patients presenting to the ED for a primary diagnosis of diabetes, a majority of the most common secondary diagnoses were no different from those conditions noted before the storm. However, there were a substantial number of patients with a secondary diagnosis of postprocedural status. This finding suggests that diabetic patients who recently had a significant surgical or medical procedure experienced difficulty in accessing appropriate follow-up aftercare, and better planning is required for these patients in the face of a pending disaster.^32^ ^33^ In a post hoc analysis, we did not find that there was a statistically significant increase in the number of emergency visits for a primary diagnosis of diabetic ketoacidosis or hyperglycemic hyperosmolar syndrome. However, this subanalysis was limited by a small number of events in the highest risk evacuation zone.

In addition, when analyzing patients with a secondary diagnosis of diabetes who presented in increased numbers for care in the week following Hurricane Sandy's landfall, we found that there was a substantial increase in the number of patients who had a primary diagnosis of myocardial infarction, hypertension, respiratory conditions, kidney disease, dialysis dependence, and prescription refills. In prior studies, patients with respiratory conditions and kidney conditions were known to be at high risk during disasters as some are ventilator dependent or dialysis dependent.^34–36^ Following Hurricane Sandy's landfall, electrical outages and damage to infrastructure led to the loss of the ability to use durable equipment at home and the closure of dialysis centers.^37–39^

In addition, the closure of outpatient clinics and hospitals with their associated medical care centers might further limit the access to regular medical care.^40^ An intervention to address the need for unfilled prescriptions may come in the form of mobile pharmacies that can deliver diabetic medications to patients in need.^41^ An important window of opportunity may exist as patients with a primary diagnosis of diabetes presented somewhat delayed on days 4 and 5 following Hurricane Sandy's landfall compared to other patients, for example, ventilator-dependent or dialysis-dependent individuals, who came to the ED for care immediately after the storm.^9^ ^42^

Prior disaster literature has documented the increased incidence of acute myocardial infarction in the general population after disasters such as Hurricane Katrina, and the Hanshin-Awaji and Niigata-Chusetsu earthquakes.^43–45^ The proposed mechanism of this increase in acute myocardial infarction is thought to be related to the mental stress and physical activity induced by disaster-related damage to property, delays in recovery and reconstruction, loss of livelihood, all coupled with disrupted access to healthcare and medications leading to an increased emotional distress, blood pressure, and catecholamine release.^44^ ^46^ Our data, although based on small numbers of events in the level 1 evacuation zone, demonstrate three times the number of cases of myocardial infarction among diabetic adults, which suggests the need to optimize cardiovascular health and to provide social support for diabetic patients highly affected by a disaster like Hurricane Sandy.^47^

Finally, in our geographic analysis of patients from all of New York City, we found that statistically significant increases in ED use primarily were concentrated in flood-prone areas. These hot spots may be areas in which interventions can be focused and geographically targeted.^48^ Furthermore, finding statistically significant clusters of diabetic patients in disaster-prone regions using geospatial analysis may help in preplanning to identify areas in which interventions may be targeted to provide home care services for patients with diabetes who had recent surgical or medical interventions, or mobile pharmacies to deliver diabetic medications, or other interventions to optimize cardiovascular health of diabetic patients at higher risk for myocardial infarction.^49^ In fact, our prior studies demonstrate that geographic analysis of patients with chronic disease and ED use can help enhance disaster planning and response to optimize care for particularly vulnerable populations of patients.

### Limitations

Our study is a geographic analysis of ED administrative claims data, which means that it is subject to coding errors that can occur in data collection. In addition, we used municipal administrative evacuation zones developed post hoc based on the damage incurred by Hurricane Sandy. These areas may not precisely represent the true underlying geographic impact of Hurricane Sandy, particularly for electrical failures. Given the number of ICD-9 diagnosis code categories that we examined, we made adjustments to the level of statistical significance required to identify a category with increased ED utilization. Important categories of ED utilization may have been missed by using this stringent requirement for statistical significance. Finally, our study is limited to Hurricane Sandy and New York City, a unique and densely populated urban environment. Findings of our study may not be generalizable to other regions of the country or other types of disasters in which different changes in ED utilization may occur.

### Conclusions

Our findings suggest that there is need to support diabetic adults during disasters by ensuring access to medications, aftercare for patients following a recent procedure, and optimize their cardiovascular health to reduce the risk of heart attacks. This study fills the void on a number of questions: specifically what conditions cause diabetic patients to present for emergency care after a disaster, and which patient characteristics among diabetic adults contribute to a higher risk of requiring acute medical needs after a disaster. In addition, this study demonstrates that geographic analysis can help identify diabetic patients who are most vulnerable after a disaster, which may help targeting interventions to improve disaster preparedness or response. The implications of these results will help shape future disaster management and policies, especially for diabetic patients, who we find are particularly at risk during events such as Hurricane Sandy.
